# Rethinking Mitchell's Chemiosmotic Theory: Potassium Dominates Over Proton Flux to Drive Mitochondrial F_1_F_o_-ATP Synthase

**DOI:** 10.1093/function/zqac012

**Published:** 2022-03-09

**Authors:** Edoardo Bertero, Christoph Maack

**Affiliations:** Comprehensive Heart Failure Center (CHFC), University Clinic Würzburg, Würzburg, Germany; San Martino Policlinic Hospital, University of Genova, Genova, Italy; Comprehensive Heart Failure Center (CHFC), University Clinic Würzburg, Würzburg, Germany; Department of Internal Medicine 1, University Clinic Würzburg, Würzburg, Germany

A Perspective on “ATP synthase K^+^- and H^+^-fluxes drive ATP synthesis and enable mitochondrial K^+^-“uniporter” function: I. Characterization of ion fluxes” & “ATP synthase K^+^- and H^+^-fluxes drive ATP synthesis and enable mitochondrial K^+^-“uniporter” function: II. Ion and synthase flux regulation”

**Figure 1.**
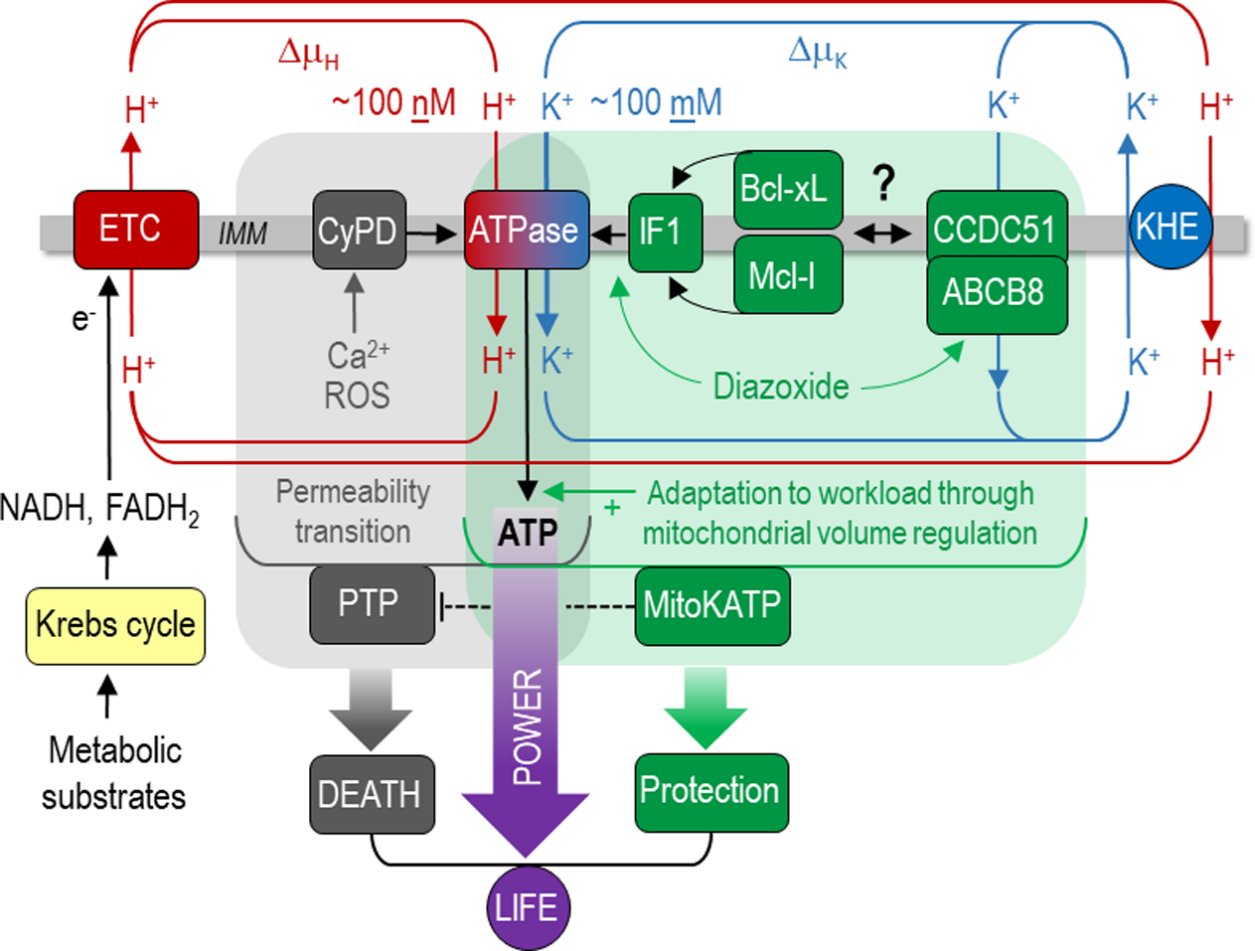
The Janus-faced mitochondrial F_1_F_o_-ATP synthase as the master regulator of life and death. The electron transport chain (ETC) receives electrons from NADH and FADH_2_ to translocate protons (H^+^) across the inner mitochondrial membran (IMM) to provide the driving force for the F_1_F_o_-ATP synthase to produce ATP. The current studies^3^^,^^6^ suggest that in addition to the H^+^ motive force (∆μ_H_), the even greater K^+^-motive force (∆μ_K_) is harnessed to drive ATP production at the ATPase. Through its impact on mitochondrial volume, this optimizes ATP production during increased ATP demand. Pathological concentrations of Ca^2+^ and/or reactive oxygen species (ROS) trigger cyclophilin D (CyPD) binding to the ATPase and thereby the formation of a permeability transition pore (PTP), which can induce cell death^9^. K^+^ flux via the ATPase is under the control of survival-related protein Inhibitory Factor 1 (IF_1_), which in turn is regulated by the Bcl-family proteins Bcl-xL and Mcl-1, to constitute a mitochondrial ATP-dependent K^+^ current (K_ATP_) that protects against PTP opening during ischemia/reperfusion and other stress conditions. Organ protection during ischemia/reperfusion provided by the canonical mK_ATP_ activator diazoxide requires IF_1_. K^+^ influx via the ATPase is counterbalanced by K^+^ extrusion via the K^+^/H^+^-exchanger (KHE). In addition to the ATPase, also CCDC51 complexing with ABCB8 constitutes functional mK_ATP_^16^, but without coupling to ATP production.

Mitochondria are the dominant source of energy in the form of adenosine triphosphate (ATP) in most cells. In the mitochondrial matrix, the Krebs cycle is fueled by nutrients to reduce nicotinamide- (NADH) and flavin adenine dinucleotide (FADH_2_)^1^, which donate electrons to the respiratory chain (Figure 1). The ensuing electron transfer along complexes I-IV of the chain and onto oxygen (O_2_) provides the energy to pump protons (H^+^) from the matrix to the intermembrane space, generating a chemical (∆pH) and an electrical potential (∆Ψ_m_) across the inner mitochondrial membrane (IMM), which together constitute the protonmotive force (∆μ_H_). According to the chemiosmotic theory developed by Peter D. Mitchell, ∆μ_H_ is the driving force for oxidative phosphorylation of adenosine diphosphate (ADP) to ATP at the F_1_F_o_-ATP synthase (Figure 1)^2^. This concept, for which Mitchell was awarded the Nobel Prize for Chemistry in 1978, has been accepted for more than 50 years and can be found in literally every textbook of biology.

In the current issue of *Function*, Juhaszova and colleagues^3^ substantially challenge—or rather expand, but do not tumble—this concept in revealing that in addition to H^+^, potassium ion (K^+^) flux through the F_1_F_o_-ATP synthase (working the same way as H^+^) provides the majority of energy to produce ATP (Figure 1). Why was this was overlooked for more than six decades? Presumably because the F_1_F_o_-ATP synthase has a > 10^7^-fold selectivity for H^+^ over other cations.^4^ But what was not sufficiently considered is that due to the 10^6^-fold higher cytosolic concentration for K^+^ (∼100 mM) than for H^+^(∼100 nM), such that K^+^ flux—driven mostly by the same high electrical driving force (∆Ψ_m_) - could be comparable to H^+^ flux via the ATP synthase.

Employing a variety of experimental systems, including proteoliposomes containing purified mammalian F_1_F_o_-ATP synthase, planar lipid membranes, but also intact rat cardiac mitochondria, Juhaszova et al.^3^ elegantly demonstrate that for each H^+^, 2.7 K^+^ ions are transferred at the ATP synthase under physiological conditions. Since contraction of intramitochondrial volume hinders the activity of the respiratory chain, and K^+^ influx osmotically allows water to expand the matrix, such two-ion flux through the F_1_F_o_-ATP synthase not only increases ATP synthesis, but also improves its efficiency: Compared to H^+^ flux, K^+^ flux exhibited a 3.5-fold higher ATP synthesis, but only a 2.6-fold higher O_2_ consumption rate.^3^ Although a “two-ion theory of energy coupling” was proposed previously by Nath,^18^ the models differ substantially: while Nath proposed a H^+^/K^+^*anti*port within the F_1_F_o_-ATP synthase may maintain electroneutrality,^18^ the model presented here^3^ defines a H^+^/K^+^*sym*port via the ATP synthase, where K^+^ extrusion is accounted for by the distinct K^+^/H^+^ exchanger (KHE; Figure 1). Importantly, this novel concept, which allows an optimized matching of energy supply to demand, was corroborated by a minimal computational model comprising the “core” mechanism constituted by ATP synthase, driven by both H^+^- and K^+^-motive force, respiratory chain, adenine nucleotide translocator, phosphate carrier, and the K^+^/H^+^ exchanger in a parallel study published elsewhere.^5^

As if this discovery was not enough of a scientific earthquake, in a second manuscript, the same authors^6^ uncover that by this previously unrecognized K^+^ flux, the F_1_F_o_-ATP synthase is also a major candidate for the long-sought mitochondrial ATP-dependent K^+^ channel (mK_ATP_), which is a central downstream effector of a phenomenon termed *ischemic preconditioning*, where repetitive brief episodes of ischemia and reperfusion of an organ reduce necrosis after a subsequent longer phase of ischemia with reperfusion by delaying the opening of the mitochondrial permeability transition pore (mPTP),^7^^,^^8^ an event that dissipates the mitochondrial membrane potential and—if nonreversible—induces cell death.^9^

K_ATP_ channels, located on the sarcolemma and the IMM, are controlled by the metabolic state of a cell: when the cellular ATP/ADP ratio drops, activation of sarcolemmal K_ATP_ channels hyperpolarizes the cell membrane, reducing its excitability to reduce ATP *demand*,^10^ whereas activation of mitochondrial K_ATP_ channels (mK_ATP_) optimizes ATP *production* through mitochondrial volume regulation,^11^ as described above. Although after its first description in the early 1990s^12^, the electrophysiological and pharmacological properties of the mK_ATP_ were extensively characterized,^11^ its molecular identity has long remained elusive. It was initially proposed that, akin to its sarcolemmal counterpart, mK_ATP_ comprised K^+^-selective pore-forming subunits from the Kir6.x family; however, this model was discarded as genetic ablation of Kir6.x channels did not suppress mK_ATP_ responses.^13^ Subsequently, the renal outer medullary K^+^ channel (ROMK) evolved as a potential pore-forming subunit of mK_ATP_ based on a proteomic screen and in vitro evidence,^14^ but cardiac-specific knock-out of ROMK later revealed that it is dispensable for cardioprotection and mK_ATP_ responses.^15^ Recently, a protein with previously unknown function (CCDC51) was identified to form a channel with mK_ATP_-like properties when associating with the ATP Binding Cassette protein 8 (ABCB8),^16^ which had already been shown to modulate mK_ATP_ activity.^17^ Since knock-out of *CCDC51* in vivo confirmed its essential role to regulate mitochondrial function in unstressed conditions and protect from necrosis during ischemia/reperfusion,^16^ CCDC51 and ABCB8 are currently the most accepted candidates in the field to constitute the mK_ATP_ (Figure 1).

The second study by Juhaszova et al.^6^ in this issue of *Function* delineates the endogenous and exogenous regulation of the F_1_F_o_-ATP synthase in its function as a K_ATP_ channel. The survival-related protein Inhibitory Factor 1 (IF_1_) is regulated by Bcl-family proteins, in particular Bcl-xL and Mcl-1, but not Bcl-2, through interaction at a BH3-like domain, which increases chemo-mechanical efficiency of the F_1_F_o_-ATP synthase to function as mK_ATP_ (Figure 1).^6^ Furthermore, the cardioprotective effect of diazoxide, the canonical mK_ATP_ activator, is shown to be mediated by IF_1_. By applying Bayesian phylogenetic analysis, the authors conclude that IF_1_ is likely an ancient Bcl family member that evolved from bacteria resident in eukaryotes and prevents excessive ATP consumption through the reversal of the ATP synthase to maintain the protonmotive force.^6^

The authors need to be applauded for providing groundbreaking results with fundamental implications for cellular bioenergetics and survival. First, these observations identify K^+^ import via the F_1_F_o_-ATP synthase as one central mechanism by which the rate of ATP turnover in the cytosol is matched by ADP phosphorylation in mitochondria. Second, they assign the F_1_F_o_-ATP synthase a central role in cardioprotection, where mitochondrial K^+^ influx via the F_1_F_o_/K^+^ uniporter elevates the threshold to elicit ROS-induced permeability transition. Of note, by coupling mitochondrial K^+^ influx to ATP production, the subsequent K^+^ extrusion via the KHE at the expense of protonmotive force is energetically counterbalanced, which is not the case when K^+^ enters mitochondria via CCDC51/ABCB8 (Figure 1), thereby avoiding the production of futile heat through “uncoupled” K^+^ leak. This led the authors to suggest that CCDC51/ABCB8-related K^+^ flux may play a rather “fine-tuning” role compared with ATP synthase-dependent mK_ATP_. However, since CCDC51 knock-out prevented most (but not all) of the cardioprotection provided by diazoxide,^16^ the herein suggested role of the ATP synthase as mK_ATP_ still needs to stand the in vivo test (of time), for instance in mice deficient of IF_1_.

In ancient Roman myth and religion, Janus is the god of beginnings, transitions, duality and endings, deciding over war and peace, or translated to biology—over life and death. Since in the past decade, the F_1_F_o_-ATP synthase has already evolved as a central component of the mPTP under the control of cyclophilin D,^9^ the novel data presented in this issue of *Function*^3^^,^^6^ deservedly assign the mitochondrial F_1_F_o_-ATP synthase a title as a *Janus-faced* enzyme, since in addition to its canonical role to produce ATP—utilizing both H^+^ and K^+^ flux—and its increasingly recognized role as a component of the mPTP,^9^ it also accounts for the protection from permeability transition through its novel function as an mK_ATP_ channel (Figure 1).
